# From bathing standards to lived waterscapes: Why we need to rethink inland blue space quality

**DOI:** 10.1007/s13280-026-02393-z

**Published:** 2026-04-13

**Authors:** David M. Oliver, Tony Robertson, Anna Wilson, Paul Hibbard, Jen Dickie, Nick Hanley

**Affiliations:** 1 Biological and Environmental Sciences, Faculty of Natural Sciences, University of Stirling, Stirling, UK; 2 School of Health and Wellbeing, University of Glasgow, Glasgow, UK; 3 School of Education, University of Glasgow, Glasgow, UK; 4 Division of Psychology, Faculty of Natural Sciences, University of Stirling, Stirling, UK; 5 School of Biodiversity, One Health and Veterinary Medicine, College of Medical, Veterinary and Life Sciences, University of Glasgow, Glasgow, UK

**Keywords:** Blue-health, Health-related outcomes, Multisensory characteristics, Recreational water quality, Wellbeing

## Abstract

Currently, the main way that recreational blue space quality is monitored and communicated to potential users is through designated bathing water status, which is evaluated via microbiological standards and epidemiological evidence; however, our experiences of blue spaces extend beyond bathing and microbiology. Interactions across sensory and ecological dynamics are central to recreation in blue spaces where perceived cleanliness, aesthetic appeal, and multisensory richness influence patterns of use, enjoyment and wellbeing outcomes. We propose a need to reconceptualise what we mean by recreational water quality, to better reflect the diversity of ‘qualities’ associated with inland blue space and their influence on health-related outcomes. In this perspective, we outline why a reconceptualisation of recreational water quality classifications is needed, suggest what could be done to work towards a reconceptualised approach of classifying and communicating recreational inland water quality and consider which institutional mechanisms could support such a shift in approach.

## Introduction

Inland blue spaces—lakes, rivers, and canals—are increasingly linked to health-related outcomes that span a range of physical, emotional, cognitive, and social benefits. Whether in or *beside* water, our immersion in blue spaces can have profound effects on our social interactions and mental health that are recognised within a wider set of ‘blue-health’ benefits (White et al. 2020). These blue-health benefits are diverse, including reduced stress and improved mood (Vert et al. 2020), increased physical activity (White et al. 2024), improved memory and attention (Kaplan and Kaplan 1989), and reduced boredom (O’Dea et al. 2025).

But not all blue spaces look and feel the same and the beneficial effects of such spaces depend on the health of the spaces themselves. Our embodied experiences of blue spaces thus depend on the health and biodiversity of the broader environment that the water supports, as evidenced by the rapidly growing evidence base in this research field (White et al. 2020; Li et al. 2023; Grace et al. 2024a). This builds on well-established pathways identified in green space research, which have demonstrated associations between contact with biodiverse natural environments and a wide range of physical, psychological, and social health benefits, from reduced cardiovascular risk to enhanced social cohesion (Aerts et al. 2018; Marselle et al. 2021; Uebel et al. 2021, 2025). Similar mechanisms are now being explored in relation to blue spaces, suggesting that ecological quality not only shapes aesthetic and sensory experience but also mediates the restorative and relational benefits derived from aquatic environments (McDougall et al. 2020; Potter et al. 2023). Consequently, the health value of blue spaces cannot be disentangled from their ecological condition.

Water quality in inland blue spaces is highly variable across both spatial and temporal scales, influenced by catchment dynamics, seasonal cycles, and human activity (Yue et al. 2023; Schauer et al. 2025). These fluctuations, in turn, shape the sensory qualities through which people experience and interpret aquatic environments. The sensory quality of blue spaces varies widely (Luo et al. 2021), depending on the dynamic integration of visual characteristics (including calming colour palettes, moderate visual complexity, open spatial configurations, and gentle motion cues), the acoustic signatures of water and wildlife, ambient scents, the multisensory experiences of wind, mist, and sunlight, and the embodied experience of movement besides, in and on water (Fisher et al. 2021; Lin et al. 2024; Guan et al. 2025).

Interactions across sensory and ecological dynamics are central to recreation in blue spaces where perceived cleanliness, aesthetic appeal, and multisensory richness influence patterns of use, enjoyment and wellbeing outcomes (Luo et al. 2023; Gilburn 2025). Yet, it is the bathing water classification system that largely determines how the recreational worth of blue spaces is judged (Tiwari et al. 2021), leaving other recreational uses and broader indicators of blue space quality unaccounted for. This is particularly problematic given that most inland blue spaces support a diversity of recreational activities, while only a small number are formally designated as bathing waters, leaving no clear mechanism for assessing or communicating their overall recreational quality. Our perspective therefore offers a view on the need to reconceptualise what we mean by recreational water quality, to better reflect the diversity of ‘qualities’ associated with inland blue space and their influence on health-related outcomes that extend beyond those typically only associated with immersive primary contact recreation (e.g. swimming). Here, we outline why a reconceptualisation of recreational water quality classifications is needed, suggest what could be done to work towards a reconceptualised approach of classifying and communicating recreational inland water quality and consider which institutional mechanisms could support such a shift in approach.

## Why reconceptualise inland recreational water management?

Currently, the main way that recreational blue space quality is monitored and communicated to potential users is through designated bathing water status (or an equivalent), evaluated via concentrations of microbial compliance parameters in the water environment (Oliver et al. 2024). Regulatory thresholds of microbial compliance parameters, known as faecal indicator organisms (FIOs), exist for primary contact recreation and are used around the world to limit risks to human health from exposure to poor water quality. These regulations draw on epidemiological evidence enshrined within the World Health Organisation (WHO) guidelines for recreational waters, which established FIO standards for primary contact recreation involving direct immersion in water (Kay et al. 2004).

*Escherichia coli* and intestinal enterococci are the most common FIOs used; they originate from many sources, including sewage, wastewater discharges, agricultural livestock, wildlife, birds, and road drainage (Seymour and McLellan 2025). Despite heightened public awareness of sewage pollution and a growing appreciation of the risks from inappropriate wastewater discharge to freshwater ecosystems, increased numbers of people are choosing to explore and value local environments as a resource for promoting their health and wellbeing (Whitty et al. 2022). However, inland blue spaces are susceptible to FIO pollution from anthropogenic inputs, with impacts exacerbated by climate change, and this can result in a ‘poor’ bathing water classification (Krupska et al. 2024). While clearly important for protecting human health, the bathing water classification system risks communicating a very narrow definition of water quality; classifying recreational water quality via microbiological compliance parameters to indicate its suitability for bathing not only underrepresents waterside users who do not swim, but also neglects how blue space quality can and does influence social and mental wellbeing. For example, a poor bathing water quality classification is associated with limited opportunities for swimming and advises against primary contact recreation, but that same blue space can still provide a range of wider blue-health benefits to other recreational users who engage in other types of water-based recreation. This might include waterside ‘exposure’ to blue spaces from jogging or birdwatching (Gilburn 2025), or secondary (limited) contact water sports such as wading, boating, and fishing where the likelihood of total immersion or water ingestion is low (Dorevitch et al. 2011). These broader blue-health benefits experienced by other recreational users are not impacted by poor microbiological water quality in the same way as for those who seek health-related outcomes from swimming; however, those wider benefits are not formally recognised and the visual signage communicating poor bathing water quality and advising against bathing further contributes to public perceptions of degraded blue space quality.

Using the United Kingdom (UK) as an example, where currently most bathing waters are coastal, the number of rivers being designated as bathing waters is gradually increasing, driven in part by calls from campaign groups to improve the ecological and recreational quality of river corridors (Oliver 2025). While this helps to distribute blue-health opportunities beyond coastal communities and provide further impetus to help tackle sewage overflows as part of river clean-up efforts, it is a form of recreational zoning that continues to prioritise swimmers over other recreational users via communication of a very narrow definition of blue space quality (Delamare et al. 2024). Moreover, relative to other bathing water environments, rivers have recorded disproportionately higher rates of failure against the minimum water quality standards considered safe for public access (Defra 2024), further eroding trust in the safety and quality of these spaces and potentially discouraging engagement with river environments despite their potential to deliver wider blue-health benefits. A broader classification of the wider-ranging multisensory ‘qualities’ of recreational inland blue space would offer a more inclusive approach for promoting the health benefits of access and exposure to these environments and would align with the growing recognition and evidence of the benefits that arise from links between health, place, and inland blue space (Grace et al. 2024b). This is especially important when considering that 50% of the world population resides within 3 km of a freshwater blue space (Ji et al. 2025).

More formal recognition of blue space qualities beyond bathing water standards would also help to address existing inequalities in access to inland waters considered suitable for recreation (i.e. those with a bathing water designation). At present, the bathing water designation system privileges a small number of inland sites, allocating a ‘quality’ classification for the purposes of immersive recreation, while leaving the majority of inland blue spaces unacknowledged and undervalued in recreational policy and public perception. Blue-health is increasingly seen as a significant benefit of ‘green infrastructure’ and an important outcome of the ‘Natural Health Service’ provided by the environment (de Bell et al. 2024; Fleming et al. 2024), but these wider benefits (delivered by pathways such as psychological restoration, social cohesion, and a strengthened sense of connection to nature) are not accounted for or recognised by bathing water classifications. Rather than designating relatively few inland waters for bathing and reporting solely on their microbiological quality for a limited subgroup of recreational users (i.e. swimmers), a more equitable and inclusive approach benefitting a much larger population would be to promote awareness of the broader benefits of recreational access to inland waters—whether rivers, canals, or lakes—and their associated waterside spaces. Emphasising wider benefits, rather than concentrating recreational activity at a small number of designated bathing sites, would also mitigate potential overcrowding and associated management challenges (e.g. Brockington et al. 2023). This further supports the move towards a multidimensional metric of blue space quality, helping to distribute recreational user groups, avoid conflicts among different user groups, and underpin more effective management of the trade-offs among recreational pursuits at inland blue spaces.

## How might we reconceptualise the classification of inland recreational water quality?

While targets and classifications for ecological and chemical status of waterbodies exist, and increased knowledge of pollutants has improved regulation (Hannah et al. 2022), these fail to recognise the multisensory qualities of waterbodies and waterside environments (e.g. acoustics, visual complexities, smells) or to connect well with public understanding due to complex legislation and terminology. Importantly, the intention of our perspective is not to prescribe a definitive reconceptualisation of how to measure and communicate inland recreational water quality, but to encourage debate on how future frameworks might better reflect the multisensory and health-related dimensions of blue space quality. This recognises that despite advances in understanding, the evidence base is not yet sufficient to warrant a complete reclassification without addressing some key research needs.

Health-related outcomes from exposure to blue space are influenced by water quality, visual appeal, and the surrounding soundscape and smellscape. Assessing blue space ‘quality’ should therefore consider these sensory dimensions together, reflecting how people perceive and engage with their environment (Ban et al. 2025; Zhai et al. 2025). Measures of quality should also include both the aquatic and terrestrial elements within which the recreational water is situated (Mishra et al. 2020). Both the terrestrial and aquatic components of blue spaces contribute acoustic and olfactory elements to the overall sensory environment, forming a soundscape and smellscape characteristic of nature (e.g. soil, water, vegetation, animals, wood, flowers, waste). These sensory cues can affect visitors’ emotional responses (Parker et al. 2024; Ji et al. 2025). While it is increasingly recognised that soundscapes and smellscapes influence health and wellbeing, there remains greater uncertainty in understanding of the links between sensory exposure and outcomes compared to visual aesthetics, where objective measures of environmental quality are more established (Bentley 2023; Huang et al. 2026). This uncertainty arises partly because links between odours and health-related outcomes vary across sociocultural contexts (Hickman 2021), and because the interactions between competing sound sources and human perceptual responses remain poorly understood (Huang et al. 2026). Developing a framework that captures how sensory factors such as sound, smell, and aesthetics influence recreation beyond conscious perceptions of water quality would be valuable, though incorporating multisensory experiences into such a framework will undoubtedly be challenging (Ban et al. 2025).

Existing frameworks for understanding soundscapes and smellscapes provide valuable insight into how sensory experiences shape human perception, engagement, and wellbeing. Schafer’s (1977) soundscape framework demonstrated how acoustic environments influence experience and provided the conceptual foundation for subsequent research linking natural soundscapes to wellbeing benefits and restorative potential. Building on this foundation, methods for assessing perceived soundscape quality have been developed to identify key attributes, establish agreed measurement scales, and define high-quality soundscapes (e.g. Axelsson et al. 2010). Porteous’s (1985) smellscape framework provides an olfactory equivalent of landscapes and soundscapes, offering a way to understand how smells of the natural environment influence sensory engagement and wellbeing, with spatially distributed odour patterns shaping how places are perceived and remembered. We propose that such frameworks offer a conceptual and methodological foundation that could be integrated within recreational water quality assessment to create a more inclusive, multisensory understanding of blue space quality. Existing visual landscape assessment frameworks, such as the Landscape Institute’s visual impact guidelines and scenic quality assessment methods (Wilson 2002), already provide structured ways to characterise light, reflection, and vegetation–water interactions, while Kaplan and Kaplan’s (1989) preference framework highlights how visual attributes such as coherence, complexity, mystery, and legibility underpin restorative responses to blue space environments. Embedding soundscape and smellscape considerations with visual, ecological, and recreational factors into water quality evaluation would help to capture a broader range of attributes that contribute to the restorative, aesthetic, and social benefits of inland waters.

Advancing this agenda means, in part, devising new theoretical and practical constructs that recognise water’s multifaceted value through which holistic assessments of blue space quality can be made. Developing inclusive and cross-cultural measures that can be understood without scientific expertise requires interdisciplinary effort to reveal the multitude of stimuli that influence societal perceptions and experiences of blue space quality. Conceptually, this includes aligning diverse methodological approaches to capture the sensory, ecological, and experiential dimensions of blue space quality within a coherent evaluative framework. At present, our understanding of the interactions between blue spaces, place, health, and wellbeing remains constrained by limited integration across disciplinary boundaries, which is a challenge that stems from different philosophical assumptions, methodologies, and communication practices (McDougall et al. 2020). To help characterise where those advances in understanding are needed, future research effort needs to: (1) improve understanding of multisensory blue space experiences, including how different sensory modalities combine to shape perception and engagement; (2) examine how specific blue space ‘qualities’ influence health and wellbeing outcomes, identifying the mechanisms and contexts through which benefits are realised; and 3) assess the trade-offs between ecological, aesthetic, economic, and social values associated with different qualities of blue space, to inform more balanced and equitable management approaches (see Box 1 for examples of research mapping to these themes). How policy shifts and institutional mechanisms influence these research needs also represents an important direction for future work.

Ultimately, a key challenge for future blue space management lies in determining how diverse health-related outcomes can be combined and weighted to reflect the overall value of inland waterbodies. Traditional assessments focus narrowly on microbiological safety, yet blue spaces also generate mental restoration, social connection, and broader wellbeing benefits. A multi-criteria assessment framework could integrate these dimensions, drawing on both quantitative indicators (such as water quality, accessibility, and patterns of use), and qualitative evidence (including perceived tranquillity, aesthetic appeal, and sense of place). In parallel, tools developed to evaluate urban blue spaces (e.g. the BlueHealth Environmental Assessment Tool, BEAT) could be integrated to provide additional evidence for design and development decisions (Mishra et al. 2020, 2021). Weightings might be derived through expert consensus, stakeholder consultation, or statistical analysis, producing a composite measure—such as a *Blue Space Health Index*—that captures the combined contribution of physical, mental, and social factors to blue space quality. However, alternative approaches could capitalise on an integration of blue-health indicators with ecosystem service models and frameworks (e.g. Garrett et al. 2023) as many of these explicitly incorporate cultural ecosystem services. Integrating blue space health indicators into such frameworks would help to formalise multisensory qualities of recreational waters as part of the “cultural services” domain. Such approaches offer potential to enable a more holistic understanding of blue space value and support management decisions that balance health protection with the promotion of wellbeing.

**Box 1:** Examples of future research needs to: (1) improve understanding of multisensory blue space experiences; (2) examine how specific blue space ‘qualities’ influence health and wellbeing outcomes; and (3) assess the trade-offs between ecological, aesthetic, economic, and social values associated with different qualities of blue space

(1) ***Improved understanding of multisensory blue space experiences***—integrating methods from psychology, social science, and the arts can help better understand the range of sensory stimuli associated with inland blue spaces and others have demonstrated how the creative arts can expand understanding of environmental issues (e.g. Angel et al. 2025). In the context of recreational water quality management this might include, for example, characterising the auditory and visual properties of different blue spaces and how these shape people’s neural, behavioural, and subjective responses as they are immersed in these environments. By coupling with self-reporting of their experiences, this would help to provide a multifaceted, integrative understanding of the role of inland blue spaces in promoting wellbeing.	
(2) ***Exploring how blue space qualities influence wellbeing***—climate change projections (e.g. Pedde et al. 2021) can be used to derive scenarios of future blue space usage and riverine bathing water designation and consider how different ‘catchment futures’ might relate to health and wellbeing inequalities linked to (i) environmental drivers and (ii) (in)justice in national water infrastructure. By combining creative approaches exploring the pedagogies of place with research methods from psychology and environmental science, the research community can better understand how experience and perceptions of exposure to different blue spaces of varying environmental quality differ in relation to, for example, water company strategies and their respective approaches to infrastructure management and public/private ownership.	
(3) ***Trading off value against different qualities of blue space***—by deploying national online choice experiments we can improve our understanding of economic-social-environmental trade-offs associated with water quality, wellbeing, recreational opportunities, and water bill payments. National online surveys can also collect information on individuals’ uses of inland blue spaces and combine both local- and national-level scenarios of future water quality changes. This can help to explore realities (opportunities, costs, and barriers) of implementing local, regional, and national decisions concerning blue space management in different spatial contexts, and to consider where public priorities may lie in terms of appropriate level and types of policy intervention concerning the management of blue spaces as therapeutic environments.	

## What institutional mechanisms could support change?

Agreeing a global concept of an integrated assessment framework for determining blue space quality is a long-term ambition and would require input from the international research and policy community. The challenge then would be to convert any resulting conceptual framework into an operational approach, requiring coordination between researchers, policymakers, and practitioners to establish measurable criteria, shared data standards, and mechanisms for ongoing monitoring and evaluation (Kuchenmuller et al. 2022). Further, translating such an integrated assessment framework into practice will require institutional mechanisms capable of supporting interdisciplinary collaboration, data integration, and evidence-based policy development. As discussed, the WHO *Guidelines for Safe Recreational Water Environments* underpin standards such as the Bathing Water Directive, where designated bathing status remains the primary benchmark for water quality. However, this focus on microbiological indicators, while essential for protecting human health, overlooks the wider physical, mental, and social wellbeing benefits increasingly linked to inland blue spaces. To address this, establishing a WHO Collaborating Centre dedicated to promoting a more holistic approach to recreational water management would offer significant opportunity. Such centres operate as part of an international network, supporting national health development through technical expertise, guidance, and knowledge exchange (Jakab et al. 2021). In addition, national and regional institutions responsible for environmental policy, landscape planning, and public health (such as regulatory agencies, research councils, or independent policy institutes) could provide critical oversight and ensure the rigorous development of a new *Blue Space Health Index* for assessing recreational water quality. These actors could facilitate alignment between scientific evidence, multisensory assessment methods (including visual, acoustic, and olfactory dimensions), and practical policy implementation.

Expanding future assessments to include visual, acoustic, and olfactory dimensions would inevitably increase costs, requiring new monitoring tools, staff expertise, and data management systems (c.f., Oliver et al. 2014). Institutional coordination, via shared funding, integration with existing monitoring programmes, or citizen science, could help to mitigate these challenges. However, recent evidence shows that budgets for environmental and regulatory services have been cut in many jurisdictions. For example, local authorities in England, Scotland, and Wales reduced their spending on environmental and regulatory services significantly since 2010 (Fahy et al. 2023); declining investment in environmental management and regulation may ultimately constrain the operationalisation of evidence-based standards for inland blue space quality, particularly given their intended application across extensive geographic scales and heterogeneous user populations.

In response, citizen science programmes could capitalise on growing availability of app-based technology and simple digital tools that enable citizen scientists to use smartphone cameras and microphones for recording sensory indicators. Crowd-sourced platforms such as 'Scenic-or-Not' also provide an efficient method for capturing aesthetic appeal across broad geographic areas (Grace et al. 2023). By leveraging large volumes of public ratings of landscape photographs, such platforms can generate spatially explicit indicators of perceived scenic quality. Integrating these datasets with sensory monitoring, e.g. via validation, or targeting areas of low agreement, could further enhance the evidence base for blue space quality standards. Local groups like Rivers Trusts are also well positioned to provide structure and training via their networks to expand monitoring programmes through shared costs between agencies, NGOs, and communities, combining scientific monitoring with public engagement (Collins et al. 2023).

## A policy shift in recreational water quality management?

Innovative and more inclusive measures of recreational inland blue space are needed to bridge blue-health benefits and inland water quality management. In our perspective, we have outlined why reconceptualisation matters and how future research and governance can help deliver change. Ultimately, future classification and communication of recreational inland blue space quality needs to: (i) celebrate inland blue environments for their wider blue-health-promoting potential, (ii) recognise significant regional differences in the overall environment of blue spaces and the multidimensional experience of people engaging with water environments, and (iii) embrace holistic, contextualised, and embodied experiences of blue spaces to fully recognise the enormous potential and impact of wider blue-health benefits beyond those gained from immersive activity in water.

New interdisciplinary understanding, evidence and approaches can help transform the current narrow assessment of blue space ‘quality’ to better reflect the wider range of blue-health benefits that recreational blue space can provide. To support such a transition would require generating evidence from a process of shared learning across disciplines to establish how people value and experience the multisensory qualities of blue spaces in terms of psychological rewards, wellbeing benefits and recreational opportunities from blue space encounters. This process can be informed by a growing evidence-base, which is helping to underpin objective and subjective measures of blue space quality in local and national blue space environments to fully account for their wider health-promoting potential. A blueprint to support a transformative and internationally transferable policy shift towards a broader interpretation of the value, health, and qualities of blue spaces beyond current approaches is now urgently needed, along with supporting stakeholder coordination mechanisms and accompanying cost–benefit analysis. We argue that such a shift will help us move beyond the over-simplified, traditional assessment of recreational water quality with the potential for significant policy impact across all countries that currently rely on a single microbiological metric to determine the amenity value of their recreational blue spaces, bringing benefits to a significantly broader segment of the population.
